# Vascular Remodelling and Mesenchymal Transition in Systemic Sclerosis

**DOI:** 10.1155/2016/4636859

**Published:** 2016-03-16

**Authors:** Pier Andrea Nicolosi, Enrico Tombetti, Norma Maugeri, Patrizia Rovere-Querini, Silvia Brunelli, Angelo A. Manfredi

**Affiliations:** ^1^School of Medicine and Surgery, University of Milano-Bicocca, 20900 Monza, Italy; ^2^Unit of Medicine and Division of Immunology, Transplantation and Infectious Diseases, San Raffaele Scientific Institute and Vita-Salute San Raffaele University, 20132 Milano, Italy

## Abstract

Fibrosis of the skin and of internal organs, autoimmunity, and vascular inflammation are hallmarks of Systemic Sclerosis (SSc). The injury and activation of endothelial cells, with hyperplasia of the intima and eventual obliteration of the vascular lumen, are early features of SSc. Reduced capillary blood flow coupled with deficient angiogenesis leads to chronic hypoxia and tissue ischemia, enforcing a positive feed-forward loop sustaining vascular remodelling, further exacerbated by extracellular matrix accumulation due to fibrosis. Despite numerous developments and a growing number of controlled clinical trials no treatment has been shown so far to alter SSc natural history, outlining the need of further investigation in the molecular pathways involved in the pathogenesis of the disease. We review some processes potentially involved in SSc vasculopathy, with attention to the possible effect of sustained vascular inflammation on the plasticity of vascular cells. Specifically we focus on mesenchymal transition, a key phenomenon in the cardiac and vascular development as well as in the remodelling of injured vessels. Recent work supports the role of transforming growth factor-beta, Wnt, and Notch signaling in these processes. Importantly, endothelial-mesenchymal transition may be reversible, possibly offering novel cues for treatment.

## 1. The Scenario: Damage and Remodelling of the Microvasculature in Systemic Sclerosis

Systemic Sclerosis (SSc) is a multisystem disease, characterized by autoimmunity, a broad microvasculopathy, and fibrosis of the skin and of visceral organs. Events still poorly characterized support the activation of myofibroblasts and self-amplifying circles lead to aberrant and sustained fibrogenesis [1]. Injury and activation of endothelial cell linings are early events in the natural history of SSc [2, 3] and excessive/deregulated innate immune responses in response to vessel and tissue injuries are hallmarks of SSc [4–8]. Vascular inflammation and remodelling characterize diverse districts, including the lung, the heart, the skin, and the kidney. Small- and medium-size arteries are usually involved, with the frequent intimal hyperplasia, medial thickening, obliteration of the lumen, perivascular inflammation, and occasionally microthrombi [9, 10] (see also below). SSc also affects capillaries. Nailfold capillaroscopy, which is routinely used in the clinical settings, often reveals dilatation of capillaries in early stages and loss in later phases, an event that possibly represents the counterpart of the lumen obliteration of small arteries in other tissues [11], a process that involves the proliferation of the intimal layer, with accumulation of constituents of the extracellular matrix [3, 12].

Of importance, the occlusion of the microvasculature results in persistent hypoxia of peripheral tissues, which in turn is not repaired by the physiologic mechanisms of vasculogenesis or angiogenesis [13]. Hypoxia represents a massive stimulus for the generation of various growth factors that influence the fate of vascular cells, prompting mesenchymal transition and fibrosis [14, 15]. On the other hand hypoxia is a key element prompting oxidative stress, another hallmark of SSc [12].

**Figure 1:**
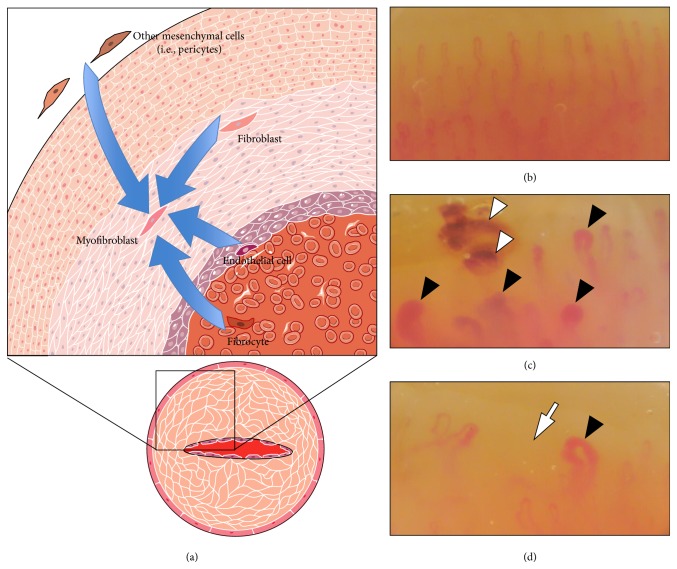
Vascular remodelling and capillaroscopic pattern in Systemic Sclerosis (SSc). (a) Stenoocclusive remodelling in SSc microvasculature (bottom right) is believed to result from an abnormal reparative attempt triggered by chronic endothelial damage, which drives intima-media hyperplasia and increased ECM production within the vessel wall. Mesenchymal cells, specifically myofibroblasts with a highly secretory phenotype, are the main final effectors responsible for these structural changes. Myofibroblasts in SSc vessels can originate from multiple cellular sources (upper left), either of mesenchymal origin, such as pericytes or fibroblast, or of nonmesenchymal origin, such as endothelial cells. (b)–(d) Capillaroscopic pattern in normal subjects (b) and scleroderma patients at magnification 200x ((c): “active” SSc pattern; (d): “late” SSc pattern). Note the heterogeneity in the architecture and morphology of SSc capillaries with frequent ectasias (black arrowheads). In the “active” scleroderma pattern there are plenty of giant capillaries (i.e., more than 50 *μ*m of diameter) and microhaemorrhages (white arrowheads), with mild loss of capillaries. In the “late” scleroderma pattern giant capillaries and microhaemorrhages are less frequent, but a severe loss of capillaries is evident, with extensive avascular areas (white arrows).

Soluble moieties present in the blood of SSc patients activate and induce in the presence of neutrophils the programmed death via apoptosis of endothelial cells [16], suggesting that inflammatory leukocytes directly contribute to the endothelial injury [17]. The apoptosis of endothelial cells [3], the aberrant expression of transcription factors [18–20], of cytokines and of growth factors, specifically including the production of the antiangiogenic VEGF165b isoform of the vascular endothelial growth factor (VEGF) [21], alterations of pathways activated by the interaction of components of the class III semaphorin family and of their receptors, Plexin-D1 and Neuropilin-1 [22, 23], and the defects of sprouting angiogenesis and vasculogenesis participate in the remodelling of the vasculature [24]. The events occurring at the cellular levels are poorly characterized. The recent insight on the relative plasticity of vascular cells, including ECs and pericytes, raises the possibility that transdifferentiation programs are activated and contribute to the maladaptive remodelling characteristic of the SSc vasculature (Figure 1).

Platelets are critical players in vascular remodelling. As guardians of the integrity of the vessels platelets respond to the early changes of the endothelial lining undergoing a burst of activation, which become persistent and sustained activation [25–27]. They represent a source of VEGF, which acts on endothelial cells [28]. Moreover they generate and release an array of profibrotic signals, including transforming growth factor-*β* (TGF-*β*), platelet-derived growth factor (PDGF), and serotonin [15]. Thrombosis of microvessels is frequent in SSc and could be facilitated by the release from damaged and activated endothelial cells of extralarge multimers of von Willebrand Factor (vWF) [29]. Platelets also contain a substantial amount of the High Mobility Group Box-1 (HMGB1) protein [30], a prototypic Damage Associated Molecular Pattern (DAMP) [31].

HMGB1 is a key signal shaping the characteristics of the inflammatory response elicited in response to sterile and microbial insults [32, 33]. It mediates the homeostatic response to injury [34–37], prompting fibrogenesis in response to endothelial damage [38–41] and playing a nonredundant role in the remodelling of vessels that takes place in injured tissues [42, 43]. Blood levels of HMGB1 are elevated in patients with SSc [44]. Conversely platelets of SSc patients undergo the depletion of the intracellular HMGB1 content [45]. The two events possibly reflect the generation of HMGB1^+^ microparticles (*μ*Ps), an event that seems to dominate the release of the molecule from activated platelets [46, 47].

Platelets are an established source of *μ*Ps and platelet-derived *μ*Ps in the plasma of patients with SSc are abundant [48, 49]. *μ*Ps have various actions that might be involved in the natural history of the disease, including the regulation of the survival and of the activation state of endothelial cells and, importantly, of endothelial cell precursors [50, 51]. Moreover, subpopulations of *μ*Ps might be associated with specific features of SSc, including lung involvement and the extent of fibrosis [52]. HMGB1^+^ *μ*Ps purified from SSc patients, but not HMGB1^−^ *μ*Ps purified from control subjects, activate human leukocytes while HMGB1 inhibitors reverse the effects* in vitro*, suggesting that the moiety might be important in the maintenance of the SSc vascular inflammation [46].

Of importance, HMGB1 is a redox-sensitive moiety [53]. HMGB1 contains cysteine residues in positions 23, 45, and 106 and resides in a predominantly reduced state in the nucleus and the cytosol [53–55]. Reduced HMGB1 in the extracellular environment forms bioactive complexes with the CXCL12/SDF1 chemokine and effectively triggers* in vitro* cell migration [53, 56–59]. An oxidizing environment in contrast enhances the ability of the molecule to prompt the secretion of inflammatory cytokines from macrophages and to promote autoimmunity [59–65]. Oxidative stress is a critical player in SSc, which contributes to the persistent activation of fibroblasts and of vascular cells [12, 66]. Indeed oxidation is critical for HMGB1 ability to support the activation of blood leukocytes in response to platelets- or *μ*Ps-derived signals [46] and possibly for their action on vascular cells, including pericytes [67, 68]. Platelet-derived HMGB1 is gaining increasing attention as a key moiety in intravascular immunity and in the activation/regulation of the coagulation cascade [45–47, 69, 70]. Further studies are necessary to validate the involvement of this pathway in SSc and specifically to reveal whether it might contribute to the remodelling of the microcirculation in particular. Of interest, HMGB1 has a well-characterized fibrogenic action and is an established inducer of epithelial-to-mesenchymal transition (EMT), a process that is associated with the origin of myofibroblasts from various precursors, including those associated with the vessel wall [40] (see below).

Other nonmural cells, such as fibrocytes and macrophages, might play a role in the development of the fibroproliferative vasculopathy in SSc. Circulating fibrocytes comprise bone marrow-derived cells that have both hematopoietic and mesenchymal features, endowed with a physiologic role in the physiologic wound healing [71]. Fibrocytes are increased in autoimmune conditions, including SSc [72, 73], and might play a part in tissue and vessel remodelling via multiple mechanisms, including the differentiation into activated myofibroblasts [71, 74].

Macrophages are attracting increasing attention for their role in the SSc (for recent excellent reviews, see [75, 76]). A detailed description of the role of macrophages in promoting and sustaining SSc vasculopathy is outside the scope of this work. However, several evidences support the contention that the recognition of endogenous ligands in peripheral tissues of SSc patients by macrophages might be involved in feed-forward self-sustaining amplificatory circuits of vascular inflammation and fibrosis [6, 37, 77].

## 2. Clinical Impact of SSc Vasculopathy

Although vasculopathy is present early and almost invariably during the course of SSc, clinical complications are traditionally classified mainly within either the fibrotic or the vascular components of the disease (Table 1). This classification is mainly based on histology and does not take into account the possible role of vascular inflammation and of vasculopathy in driving the fibrotic component of the disease.

Despite the fact that therapeutic improvements have changed the relative impact of SSc complications on patients' prognosis [78] SSc vasculopathy, in terms of pulmonary arterial hypertension (PAH), heart involvement, and scleroderma renal crisis (SRC), still represents the first cause of disease-related mortality. Therapeutic targets are different in the vascular complications of SSc, suggesting that the pathogenesis of these conditions only partially overlaps. SRC prognosis has fortunately much improved since the recognition of the therapeutic role of ACE inhibitors [79]. SRC is typically characterized by malignant hypertension and rapidly progressive renal failure. Organ dysfunction (hearth failure, encephalopathy, and microangiopathic haemolytic anaemia) frequently coexist [80]. Histology shows onionskin-like lesions and fibrotic intimal sclerosis, with possible adventitial fibrosis and intravascular thrombosis [81]. Pathophysiology of SSc hearth involvement is complex and heterogeneous, but vasculopathy is believed to be the most frequent mechanism, resulting in focal and patchy myocardial ischemia and consequent fibrosis with either systolic or diastolic dysfunction [82].

## 3. Pulmonary Arterial Hypertension

Pulmonary hypertension (PH) is defined as an elevated mean pulmonary arterial pressure (mPAP) ≥ 25 mmHg at rest [83]. PH is frequent in SSc and can be associated with lung and hearth involvement or thromboembolic disease. Pulmonary arterial hypertension (PAH) is a disease characterized by progressive obliterative vasculopathy involving the distal pulmonary circulation, the distal pulmonary arteries in particular [83]. Progressive precapillary PH (i.e., PH with a pulmonary capillary wedge pressure ≤ 15 mmHg and pulmonary vascular resistance > 3 Wood Units) defines PAH. This results in progressive right heart failure [84], with a median survival without therapy of about 2.8 years from diagnosis [85]. SSc is one of the main causes of PAH [83]. Currently, PAH and interstitial lung disease represent the first causes of disease-related mortality in SSc patients. SSc-associated PAH (SSc-PAH) has a prevalence between 10 and 12% of SSc patients and may occur even many years after the diagnosis [86]. SSc-PAH is associated with limited scleroderma, presence of anti-U3RNP autoantibodies, late-onset disease, multiple telangiectasias, digital ulcers, and worsening lung diffusion [86].

Mortality of SSc-PAH is worse than mortality of idiopathic PAH [87, 88]. Early detection is therefore fundamental but remains challenging. Symptoms are caused by heart failure or worsening respiratory function and occur late during disease course. The diagnosis is not based on the direct identification of the lung vasculopathy but on the indirect evaluation of its hemodynamic impact, which can be definitively assessed with right heart catheterisation only, when the lung vascular reserve is already substantially compromised [89].

With the exception of a small group of patients with hereditary or idiopathic PAH responding to calcium channel blocker vasodilators, structural remodelling of the lung microcirculation is substantial. Currently available therapies for PAH antagonise endothelin-1 (ET-1) receptors increase concentrations of prostacyclin or its analogues or increase cyclic GMP in the lung vasculature antagonising phosphodiesterase-5. All these agents are believed to target both the vasoconstriction and the remodelling observed in the lung vasculature. However, patients with SSc-PAH have a poorer response to therapies, in comparison with other PAH subgroups [88], and up-front combination regimens of oral agents antagonising ET-1 receptors and phosphodiesterase-5 may provide a more effective intervention [90, 91]. Autoimmunity with unrelenting inflammatory responses and more severe vessel and cardiac involvement might account for the poorer response to therapy of patients with SSc-PAH as compared to those with idiopathic PAH [88].

Histology of PAH is reminiscent of other small vessel vasculopathies, such as SSc. An obliterative and onionskin-like intimal and medial thickening is the pivotal finding. Intravascular thrombosis is frequent, and perivascular inflammation is observed. Muscularisation of small arteries as well as perivascular inflammation is typical. Endothelial cells may have a disorganised growth within the lumen of remodelled vessels, to form the so-called plexiform lesions [92]. SSc-PAH pathology is similar, with more abundant inflammatory infiltrates and more frequent concomitant involvement of the venous compartment of the lung circulation [93].

Mechanisms underlying these changes are poorly understood. Similar histologic features of remodelled arteries and intimal hyperplasia are not exclusive of SSc but are believed to be a stereotyped vascular response to many types of injuries. Large-vessel vasculitides such as Takayasu arteritis [94] and giant cell arteritis [95] are inflammatory conditions in which arterial remodelling and intimal hyperplasia play a central role. Similarly to SSc, in Takayasu arteritis the progression in vascular stenoocclusions and the intensity of systemic inflammation poorly correlate [96–98]. Further studies are required to verify whether molecular events regulating cell plasticity in the SSc vessel walls might have a role in macrovascular diseases.

Increased numbers of cells expressing alpha-smooth muscle actin (alpha-SMA) are a nearly universal finding in the remodelled artery. Resident smooth muscle cells have been traditionally regarded as the predominant source of the newly appearing alpha-SMA-expressing cells. However, rapidly emerging experimental evidence suggests that other sources might play a role. We will briefly discuss below the possible contribution of the Endothelial to Mesenchymal Transition (EndoMT) and the evidence supporting a role of transforming growth factor-beta, Wnt, and Notch signaling in this process.

## 4. EndoMT and TGF-**β**

EndoMT refers to a transdifferentiation process in which endothelial cells downregulate the expression of endothelial markers, such as CD31 and vascular endothelial cadherin (VE-cadherin), acquiring a mesenchymal/myofibroblast phenotype, which is characterized by the expression of SMA, collagen type I (Col I), together with Twist 1, a specific marker of mesenchymal transition [74, 99].

EndoMT has emerged as a player in the pathogenesis of tissue fibrosis in various diseases, including diabetic nephropathy, cardiac fibrosis, intestinal fibrosis, portal hypertension, and PAH [100]. Experimental evidence supports a role of EndoMT in SSc as well [100–102]. Of importance, lung tissues of patients with interstitial lung disease associated with SSc have been elegantly shown to contain cells that simultaneously express EC-specific and mesenchymal proteins and transcripts, demonstrating that EndoMT actually occurs in target organs of the disease [103]. EndoMT could contribute, under the action of signals generated by inflammatory leukocytes recruited and activated into the perivascular tissues, to the conversion of endothelial cells into activated myofibroblasts, that is, cells responsible for the formation of scar tissue and for fibrosis [74, 99]. Thus, EndoMT would causally connect two hallmarks of SSc, the aberrant fibrogenesis and the persistent endothelial injury. TGF-*β*, a cytokine involved in embryogenesis, cellular differentiation, development, and inflammatory response, plays a role in fibrotic diseases by stimulating the production of collagens and other ECM components and by inhibiting the expression of various relevant metalloproteinases. TGF-*β* is in particular a central cytokine in SSc [10]. TGF-*β*-regulated genes are expressed in the skin and the lung of patients with SSc and the extent of the cytokine expression correlates with the disease activity [10]. Moreover, mutations in the TGF-*β*-sensing ALK-1 signaling pathway cause familial PAH and hereditary haemorrhagic telangiectasia, indicating a role of TGF-*β* signaling in both SSc vasculopathy and fibrosis [10].

TGF-*β* is able to induce plasticity in endothelial cells, committing them toward a fibrogenic fate. The process involves the acquisition of a mesenchymal progenitor multipotent status and is characterized by the transient expression of PDGFR*α* mRNA, by the increase of the mesenchymal markers expression (such as *α*-SMA and Col I), and by the reduction of endothelial markers expression, CD31 and Tie 1 [104]. Li and Jimenez in 2011 observed in primary mouse pulmonary ECs the ability of TGF-*β* to induce *α*-SMA and type I collagen expression together with an inhibition of VE-cadherin. These effects were associated with an increased Snail-1 expression, involving the c-Abl tyrosine kinase and protein kinase C*δ* (PKC*δ*) activity [105].

## 5. Wnt

The Wnt proteins comprise a family of glycoproteins that via canonical and noncanonical intracellular signaling pathways play crucial roles during embryonic development. Wnt proteins and pathways have been also implicated in the pathogenesis of fibrotic diseases, including SSc [106–108]. TGF-*β* activates the canonical Wnt pathway, and multiple genes involved in tissue repair and in fibrosis are transcriptional targets of Wnt/*β*-catenin [109]. Transcriptional analysis of primary alveolar epithelial type II (ATII) cells from patients with idiopathic pulmonary fibrosis (IPF) revealed an elevated expression of genes coding for Wnt ligands, receptors, regulators, and targets [110, 111]. Other studies provided evidence of an increased Wnt expression and activity in the skin and the blood of patients with SSc [112]. Nuclear *β*-catenin, a marker of active canonical Wnt signaling, was strongly upregulated in the lung of patients with SSc-associated fibrosis [106]. Wnt3a could be implicated in the modulation of EndoMT in human dermal microvascular endothelial cells via the reduction of vascular endothelial cadherin mRNA expression and induction of vimentin and slug mRNA expression [113].

## 6. Notch-Jagged

The Notch signaling is also a fundamental pathway governing development. Notch receptors and their ligands have been located in the vascular system. Notch activation in endothelial cells results in morphological, phenotypic, and functional changes consistent with mesenchymal transformation. These changes are correlated with EndoMT, including downregulation of endothelial markers, upregulation of mesenchymal markers, and migration toward platelet-derived growth factor-BB. Notch and TGF-*β* signaling synergistically induce the Snail expression in endothelial cells. Notch activation inhibits TGF-*β*/Smad1 and TGF-*β*/Smad2 signaling pathways by decreasing the expression of Smad1 and Smad2 and their target genes. In contrast, Notch increases Smad3 mRNA expression and protein half-life and regulates the expression of TGF-*β*/Smad3 target genes in a gene-specific manner [114].

Notch signaling appears to be activated in the skin of patients with SSc, with overexpression of the ligand, jagged-1. This appears to be a nonredundant event in fibrogenesis, since genetic or pharmacological interference with this pathway inhibited the development of fibrosis in experimental animals, interfering with the generation of autoantibodies as well [115]. Thus, data in the literature suggest that the Notch pathway is correlated with EndoMT and that the same pathway is deregulated in SSc. However direct experimental evidence of Notch involvement in the modulation of EndoMT in SSc is so far missing.

## 7. Endothelin 1

Endothelin-1 (ET-1), a 21-residue peptide, is a potent vasoconstrictor. ET-1 regulates the vascular tone through interaction with endothelin receptors A (ETRA) and B (ETRB), prompts fibrogenesis, and possibly contributes to the vessel's instability and capillary rarefaction during SSc. Some* in vitro* evidence suggests that ET-1 might promote EndoMT on ECs isolated from SSc patients and macitentan, a dual endothelin-1 receptor antagonist, blocks the EndoMT induced* in vitro* by the combination of TGF-*β* and ET-1 [116]. The actual relevance of* these in vitro *observations for the SSc vasculopathy remains to be established. For example,* in vitro* studies supported an antifibrotic effect of the ET-1 receptor antagonist, bosentan, which however was not consistent upon treatment of SSc patients with PAH (e.g., see [117]).

## 8. Interferon

Interferon has also been studied in the setting of EndoMT. IFN-*α* appears to downregulate while IFN-*γ* appears to upregulate *α*-SMA, CTGF, ET-1, and TGF*β*2 expression in human dermal microvascular endothelial cells. In this* in vitro* experimental setting, the blockade of TGF*β* signaling normalized IFN-*γ*-mediated changes in Fli-1, VE-cadherin, CTGF, and ET-1 levels, whereas the upregulation of *α*-SMA and TGF*β*2 was not affected. IFN-*γ* also induced the expression of selected genes related to EndoMT, including Snail-1, FN1, PAI1, TWIST1, STAT3, RGS2, and components of the Wnt pathway [118].

## 9. MicroRNAs

MicroRNAs (miRNAs) consist of a class of small endogenous noncoding RNAs, approximately 22 nucleotides long, able to regulate posttranscriptionally gene expression. A single miRNA can modulate hundreds of target genes by suppressing translation, mediating mRNA segmentation, or causing RNA destabilization. On the other hand, multiple miRNAs can cooperate to regulate the expression of a single target gene. miRNAs might be involved in the natural history of SSc. A downregulation of miRNAs involved in the suppression of fibrosis (such as miR-29a, miR-196a, and miR-150) has been reported in SSc patients [119]. Upregulation of miRNAs able to induce the Col1A1 expression (such as miR-21b) or other ECM molecules (miR-92a) has also been reported [120]. Conversely, miR-7, a miRNA with a role in the suppression of fibrosis, is upregulated in SSc fibroblasts possibly because of a negative feedback loop, associated with thrombospondin-2 upregulation [121].

TGF-*β* significantly increased miR-21 expression in endothelial cells and induced EndoMT. Mechanistically, miR-21 acts on phosphatase and tensin homolog in endothelial cells, favoring the activation of the Akt pathway [122]. A miRNA array on mouse cardiac endothelial cells and EndoMT-derived fibroblast-like cells revealed that miR-125b, Let-7c, Let-7g, miR-21, miR-30b, and miR-195 were significantly elevated during EndoMT, while levels of several miRNAs including miR-122a, miR-127, miR-196, and miR-375 were significantly downregulated [123]. Some of these signals, such as the miR-125b, might be directly implicated in the fibroblast-to-myofibroblast transition [124]. Although miRNA modulation appears to be an interesting field that might shed light on biological events occurring in SSc, little experimental evidence supports so far the contention that miRNA modulation actually occurs in endothelial cells of SSc patients. Moreover, like for other epigenetic regulations that have been implicated in the pathogenesis of SSc, their causal role in the various disease features remains elusive [125]. Specifically, it remains to be seen whether miRNA modulation reflects ongoing EndoMT, or it is a necessary condition to begin or effectively conclude the process.

## 10. Oxidative Stress and EndoMT

Oxidative stress mediated by reactive oxygen species (ROS) plays a role in various features of SSc [12, 66, 126, 127], possibly including senescence-correlated changes of SSc fibroblasts [128] and of bone marrow-derived mesenchymal stem cells of SSc patients, which express markers of early senescence and have an impaired ability to differentiate into endothelial cells [129]. Defective function of endothelial progenitor cells might contribute to the defective angiogenesis typical of the disease [13]. The NADPH oxidase (NOX) family of membrane-associated enzymes catalyzes the reduction of oxygen to form ROS. NOX4 in particular has a key role in the establishment and maintenance of tissue fibrosis. Several signals involved in SSc pathogenesis, including TGF-*β*, PDGF, and ET-1, modulate the expression of NOX and of NOX4 in particular [130]. Oxidative stress also induces the conversion of ECs into myofibroblasts via a mechanism possibly depending on ALK5/Smad3/NF-*κ*B pathway [126].

## 11. Shear Stress

Uniform laminar shear stress (LSS) has anti-inflammatory and anticoagulant effects on ECs [131]. Conversely, EndoMT might be implicated in the fibroproliferative vascular disease and might be modulated by shear stress in a ERK5-dependent manner [131]. Prolonged exposure of EC to LSS results in sustained activation of p53 and in growth arrest [132]. KLF4 physically interacts with p53 in synergistic activation of p21, indicating interaction between p53 and ERK5 signaling pathways. Activation of ERK5 thus not only inhibits mesenchymal transition of EC, but also might be the key to reversal of the transition [131].

## 12. EMT in SSc

EMT is a process in which adhesive properties and polarity of epithelial cells are modified, with decreased expression of epithelial markers, including E-cadherin and Zo-1. In contrast expression of mesenchymal markers, such as vimentin and fibronectin, is upregulated [133] and matrix metalloproteinases (MMPs) are generated including MMP-2 and MMP-9, which degrade collagen IV, the main component of the basement membrane, and aid the development of a migratory phenotype. The retained plasticity of pulmonary and renal epithelial cells and their ability to contribute directly to human fibrotic disease via EMT are well defined [134, 135].* In vitro* data suggest the involvement of TGF-*β* and TNF-*α* synergic activity in driving EMT of primary keratinocytes, in a Smad-dependent manner. The use of specific Smad inhibitors could prevent EMT but more importantly can also reverse established EMT open to a new potential therapeutic intervention [133]. SSc keratinocytes exhibit a phenotype normally associated with tissue repair, including phosphorylation profiles indicative of TGF-*β* signaling, with increased phosphorylated Smad2/3 nuclear translocation [136].

An important role in the EMT during SSc is also played by the lacking activity of Fli-1. The transcription factor Fli-1, a member of the Ets transcription factor family, is epigenetically suppressed in SSc skin and SSc dermal fibroblasts and may represent such a predisposing factor for SSc [137]. Fli-1 expression is decreased in nonlesional SSc skin in various cell types, including dermal fibroblasts, endothelial cells, and perivascular inflammatory cells, suggesting that downregulation of Fli-1 is an early event preceding the development of fibrosis. The factors that might be involved in the downregulation of Fli-1 include TGF-*β* and interferon-*γ*, in addition to epigenetic mechanisms, and recent data suggest a new* in vivo* model to study the SSc phenotype in various cell types [138]. Indeed, bleomycin-induced skin fibrosis in Fli-1^+/−^ mice highlights alterations of dermal fibroblasts, endothelial cells, and macrophages reminiscent of the human disease, suggesting a new promising tool for the* in vivo* study of SSc [138].

## 13. Conclusions

**Figure 2:**
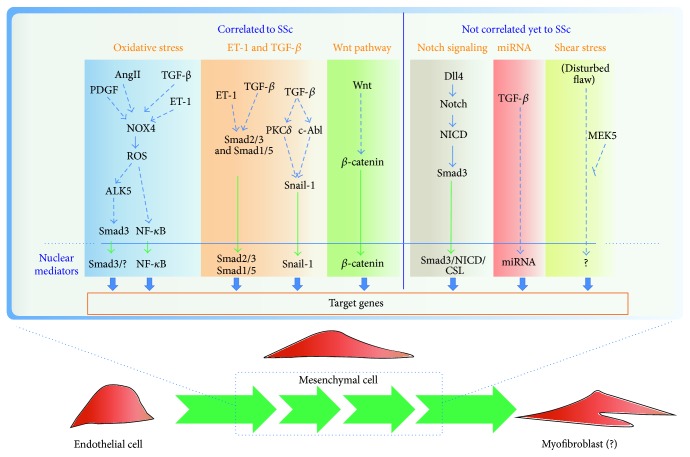
Pathways involved in the EndoMT. The scheme summarized the putative pathways involved in the EndoMT highlighting which are already correlated or not with SSc. The activation of specific nuclear mediator leads to activation of target genes that are correlated with the increase of mesenchymal markers (such as Col I, *α*-SMA, and Twist 1) and/or decrease of endothelial markers (such as CD31, VE-Cad, and Fli-1). The activation of these pathways could lead endothelial cells to acquire initially mesenchymal characteristics and later on to acquire myofibroblastic features.

A failure of various intermingled homeostatic programs accounts for the complex phenotype of SSc patients. Defective homeostatic processes are governed by interacting signaling pathways, most of which are involved in the regulation of the vascular cell plasticity (Figure 2). Although much new information has been obtained on the mesenchymal transition of endothelial and epithelial cells and on the transition from fibroblasts to myofibroblasts over the past few years, many issues still require characterization, including the actual extent to which mesenchymal transition occurs in SSc patients. The contention that cell plasticity causally links the generalized vascular inflammation and remodelling with the fibrosis associated with SSc has not been formally demonstrated. In case it was, the molecular regulation underlying the substantially variable fibrosis (generalized versus limited) which characterizes each single patient would remain to be established. Moreover the contribution of autoimmunity in the process, which is felt to be important, remains elusive. New targets for molecular treatments are being identified. These discoveries may lead to profound advances in therapies for SSc and possibly for other persistent fibrotic and inflammatory diseases.

## Figures and Tables

**Table 1:** Most prominent fibrotic and vascular complications of SSc.

Fibrotic complications	Vascular complications
Skin fibrosis	Raynaud phenomenon

Lung fibrosis	Ischemic ulcers

Gastrointestinal involvement	Acral ischaemia/necrosis
	Gastral antral vascular ectasia (GAVE) and gastrointestinal telangiectasias
	Scleroderma renal crisis
	Heart involvement
	Pulmonary arterial hypertension
